# Saliva molecular inflammatory profiling in female migraine patients responsive to adjunctive cervical non-invasive vagus nerve stimulation: the MOXY Study

**DOI:** 10.1186/s12967-019-1801-y

**Published:** 2019-02-22

**Authors:** Azize Boström, Dirk Scheele, Birgit Stoffel-Wagner, Frigga Hönig, Shafqat R. Chaudhry, Sajjad Muhammad, Rene Hurlemann, Joachim K. Krauss, Ilana S. Lendvai, Krishnan V. Chakravarthy, Thomas M. Kinfe

**Affiliations:** 1 Department of Neurosurgery, University Hospital Bonn, Bonn, Germany; 2 Department of Psychiatry, University Hospital Bonn, Bonn, Germany; 3 Division of Medical Psychology, University Hospital Bonn, Bonn, Germany; 4 Department of Clinical Chemistry and Clinical Pharmacology, University Hospital Bonn, Bonn, Germany; 5 Department of Neurosurgery, Helsinki University Hospital, Helsinki, Finland; 6 Department of Neurosurgery, Medical School Hannover, Hannover, Germany; 7 Department of Anesthesiology and Pain Medicine, University of California San Diego, San Diego, CA USA; 8 Rheinische Friedrich-Wilhelms University Bonn, Sigmund-Freud Street 25, 53105 Bonn, Germany

**Keywords:** Migraine, Saliva oxytocin/IL-1β, Cervical non-invasive vagus nerve stimulation, Trigemino-nociceptive signaling, MOXY pilot study

## Abstract

**Background:**

Rising evidence indicate that oxytocin and IL-1β impact trigemino-nociceptive signaling. Current perspectives on migraine physiopathology emphasize a cytokine bias towards a pro-inflammatory status. The anti-nociceptive impact of oxytocin has been reported in preclinical and human trials. Cervical non-invasive vagus nerve stimulation (nVNS) emerges as an add-on treatment for the preventive and abortive use in migraine. Less is known about its potential to modulate saliva inflammatory signaling in migraine patients. The rationale was to perform inter-ictal saliva measures of oxytocin and IL-1ß along with headache assessment in migraine patients with 10 weeks adjunctive nVNS compared to healthy controls.

**Methods:**

12 migraineurs and 12 suitably matched healthy control were studied with inter-ictal saliva assay of pro- and anti-neuroinflammatory cytokines using enzyme-linked immuno assay techniques along with assessment of headache severity/frequency and associated functional capacity at baseline and after 10 weeks adjunctive cervical nVNS.

**Results:**

nVNS significantly reduced headache severity (VAS), frequency (headache days and total number of attacks) and significantly improved sleep quality compared to baseline (p < 0.01). Inter-ictal saliva oxytocin and IL-1β were significantly elevated pre- as well as post-nVNS compared to healthy controls (p < 0.01) and similarly showed changes that may reflect the observed clinical effects.

**Conclusions:**

Our results add to accumulating evidence for a therapeutic efficacy of adjunct cervical non-invasive vagus nerve stimulation in migraine patients. This study failed to provide an evidence-derived conclusion addressed to the predictive value and usefulness of saliva assays due to its uncontrolled study design. However, saliva screening of mediators associated with trigemino-nociceptive traffic represents a novel approach, thus deserve future targeted headache research.

*Trial registration* This study was indexed at the German Register for Clinical Trials (DRKS No. 00011089) registered on 21.09.2016

## Background

Migraine represents a devastating primary headache disorder affecting approximately 14% of the population with an emerging prevalence and socio-economic burden [1–4]. The distinction and definition of episodic and chronic migraine has been an issue of ongoing debate [5, 6]. For instance, both have been reported to differ in prevalence, symptom profile, socio-demographics, individual/economic burden and co-morbidities [6]. However, preventive and abortive pharmacological/behavioral interventions overlap in both migraine subtypes and failed to achieve favorable response in a considerable proportion of migraineurs [6–8]. Thus, cervical non-invasive vagus stimulation (nVNS) has been approved to represent a reasonable and safe adjunctive treatment option for prevention and abortive migraine therapy [9–16]. In two RCT trials, the EVENT study (chronic migraine prevention with non-invasive vagus nerve stimulation) and PRESTO study (prospective study of nVNS for the acute treatment of migraine) and several prospective observational cohort studies, nVNS demonstrated the capability to effectively act as adjunctive prophylaxis and rescue intervention in episodic and chronic migraine as well as in associated mood and sleep disturbance [10–12, 16–19]. Further long-term follow-up observations by Martelletti and colleagues and an additional post hoc analysis confirmed the initial findings of the PRESTO study [17, 18]. Interestingly, transcutaneous stimulation of the auricular branch of the vagal nerve (t-VNS) at 1 Hz promoted a significantly larger reduction of chronic migraine frequency compared to 25 Hz t-VNS. Of note, t-VNS duration lasted for 4 h per day during the 3 months study period [19]. In order to parallel acute and chronic head pain and to investigate possible VNS-induced changes in the trigemino-nociceptive system, several preclinical studies confirmed the clinical observed VNS responsiveness, although the precise pathways are not fully understood [20–28].

Oxytocin is synthesized in neurons exclusively located within the hypothalamic nuclei (nucleus paraventricularis of the hypothalamus; PVN) and the supraoptic nucleus (SOP). Magnocellular neurons are distributed in the PVN and in the SOP and project to the posterior pituitary lobe (release oxytocin into the blood flow) and secondly, these neurons are connected with brain areas such as the amygdala, hippocampus, and cerebral cortex. A smaller population of parvocellular oxytocinergic neurons associated with the PVN interacts via receptor signaling with the brainstem and the spinal cord (dorsal column layers/dorsal root ganglion), but not via systemic blood circulation. Thus, through both pathways, oxytocin has been suspected to impact central and peripheral nociceptive transmission and neuro-inflammatory pain signaling [29–31]. Observational studies examined migraine relief after oxytocin administration in the past [32, 33]. Tzabazis and colleagues investigated the anti-nociceptive head pain potential of oxytocin in preclinical and human trials [34, 35]. Remarkably, after administration of radiographic labeled oxytocin, high concentrations were tracked in the trigeminal nucleus caudalis (TNC), the trigeminal ganglion (TG) and corresponding three trigeminal branches (V1–V3), which innervate the corresponding mucosa and glandulae [35].

Earlier reports applying VNS for depression and seizure observed peripheral changes of pro-/anti-inflammatory cytokines (IL-1β, TNF-α) in small-scale cohorts [36, 37]. Of interest, ictally elevated IL-1β concentrations were observed in the jugular blood of migraine patients [38]. Perini and et al. demonstrated intra-ictally elevated levels of IL-1β and TNF-α in migraine patients compared to healthy controls, which was re-examined in a most recent cytokine migraine study [39, 40]. In addition, nVNS significantly decreased serum concentrations of IL-1β (pro-inflammatory) and increased anti-inflammatory marker IL-10 compared to sham stimulation in healthy individuals [41]. Although not fully understood, experimental data indicate that IL-1β promotes activation of trigemino-nociception and peripheral/central neuro-inflammatory pathways involved in headache onset [42–46].

This is the first study assessing saliva oxytocin and IL-1β concentrations along with score-based assessment of clinical responsiveness [head pain severity, frequency (headache days-attacks/month), functional state (sleep quality, mood, quality of life)] in migraine patients treated with adjunctive cervical nVNS.

## Methods

### Study design

The rationale of this prospective observational case control study was to investigate the efficacy of nVNS as an adjunct to medication in patients with treatment-refractory episodic migraine (EM) and chronic migraine (CM). In addition to a variety of functional outcome measures, interictal (defined as 48 h apart from an attack) saliva concentrations of pro-inflammatory cytokine IL-1ß and anti-inflammatory oxytocin were assessed at baseline (pre-nVNS) and re-assessed after 10 weeks of adjunctive nVNS treatment (post-nVNS).

### Ethics, consent, permissions

This study was performed according to the guidelines of the latest revision of the declaration of Helsinki. Ethics approval for this study was obtained from the institutional review board (Ethic Commission University Hospital Bonn IRB no.: 296/15). All patients provided written informed consent. Furthermore, the study was pre-registered at the German Register for Clinical Trials (DRKS No. S00011089).

https://www.drks.de/drks_web/navigate.do?navigationId=trial.HTML&TRIAL_ID=DRKS00011089.

### Study population and clinical assessment

The study enrollment was from September to October 2016. The patients were assigned by a headache specialist (anesthesiologist/neurologist) to our university hospital. In addition, the diagnosis of the refractory headache disorder was confirmed from an interdisciplinary internal pain board (including a neurologist, an anesthesiologist, a neurosurgeon, psychiatrist, and pain nurse) in cooperation with a tertiary level headache center according to the criteria listed of the International Classification of Headache Disorders (ICHD; third edition; beta). In particular, the terms refractory and drug-resistant migraine are still highly debated [1, 47, 48]. The patients were refractory to four classes of preventive medication and/or experienced side-effects (β-blockers, anticonvulsants, tricyclic antidepressants, calcium channel blockers) using different dosages of rescue drugs. In addition, 10 (2 CM/8 EM) patients out of 12 patients experienced a less favorable outcome with the usage of botulinum toxin. Of note, botulinum toxin represents off label in EM and probably not effective in EM. Standard medication was stable 4 weeks prior to baseline visit according to the individual’s prescriptions and remained unchanged through the entire study period. Depending on their intensity, migraine attacks were categorized as severe (severe = VAS 7–10/10, moderate = VAS 4–6/10 or mild = VAS 1–3/10). Inclusion and exclusion criteria are outlined in Table 1.

**Table 1:** Inclusion and exclusion criteria according to the study protocol

Inclusion criteria	Exclusion criteria
Chronic refractory headache disorder according to the International Classification of Headache Disorders ICHD (third edition; beta) Age equal/greater 18 Informed consent (Study, nVNS) Refractory to medical and/or behavioural therapy Medication overuse headache has been ruled out Eligible for vagus nerve stimulation Willingness to a defined follow-up interval Intracranial and cervical pathologies excluded by MR scan Standard medication 4 weeks prior to nVNS and within the entire study period according to the individual’s prescriptions	No informed consent Other concomitant neuropsychiatric comorbidity not adequate classified and/or requiring specific diagnosis or treatment Pregnancy Malignancy Previous performed invasive, noninvasive and ablative procedure Not willing to complete pain diary regarding severity and frequency

Functional outcome measures collected at baseline (pre-nVNS) and after accomplishment of 10 weeks nVNS treatment (post-nVNS) evaluating the mean change from baseline in patient-reported head pain intensity (visual analogue scale, VAS) and frequency (mean change in number of headache days and attacks compared to baseline). Baseline values for number of headache days and migraine attacks per month were assessed on the basis of patient self-report/headache diaries and medical records Participants recorded functional outcome measures during the 10 weeks of nVNS treatment on a daily basis. In addition to patients’ self-report (headache diaries), clinical outcome measures were assessed through interviews during the outpatient visits in the 11th week after baseline in an inter-ictal period (defined as 48 h apart from an attack). Data of all reported and treated attacks within the 10 weeks of nVNS therapy were pooled and analyzed. Relevant migraine co-morbidities including impaired sleep quality (Pittsburgh Sleep Quality Index, PSQI), depressive symptoms (Beck Depression Inventory, BDI), health status (EuroQuol EQ-5D-5L), impact of headache on life (Migraine Disability Assessment, MIDAS) and Body Mass Index (BMI) [49, 50]. Pain relief was defined as a ≥ 50% reduction in severity and/or frequency of attacks.

### Demographic and baseline characteristics

Of the 14 participants initially enrolled in the study, two patients were excluded from analysis due to protocol violation, such that the final analysis included data acquired from 12 patients. One participant changed pain medication and another discontinued nVNS due to temporary skin discomfort. All patients were female with a mean age of 47.6 years (range 34–65 years). The majority presented with EM (n = 10, five with aura) and two patients presented with CM (with aura) (Table 2).

**Table 2:** Demographic data and baseline characteristics of the study population addressed to severity, frequency and current preventive and abortive medication

Patient No.	Migraine type	Number of attacks per month	Pain intensity (VAS) Score	Number of headache days per month	Prophylactic medication at baseline	Acute medication at baseline
1	CM+	10	7/10	18	None	TRIP + NSAD
2	EM+	12	5/10	12	ß-blocker + TCA	TRIP + NSAD
3	EM−	5	7/10	9	ß-blocker + TCA	TRIP
4	EM−	12	8/10	14	None	TRIP
5	CM+	16	8/10	26	None	TRIP
6	EM−	7	6/10	9	None	TRIP
7	EM+	2	8/10	8	None	TRIP + NSAD
8	EM−	11	7/10	12	None	NSAD
9	EM+	10	8/10	10	Magnesium	TRIP + NSAD
10	EM+	3	8/10	3	SSRI	TRIP + NSAD
11	EM+	8	8/10	14	ß-blocker + TCA	TRIP
12	EM−	10	9/10	14	ß-blocker	TRIP + ASS

*f* female, *VAS* visual analogue scale, *CM* chronic migraine, *EM* episodic migraine, *±* with/without Aura, *TRIP* triptans, *TCA* tricyclic Antidpressants, *SSRI* Selective Serotonin reuptake inhibitor, *NSAD* nonsteroidal anti-inflammatory drugs, *ASS* acetylsalicylic acid, *nVNS* cervical non-invasive vagus nerve stimulation, preVNS medication remained stable 4 weeks prior to study enrollment (see inclusion criteria)

Eleven patients were classified as MIDAS grade III/IV and one patient was classified as grade I. Evaluation of sleep patterns at baseline revealed that 10/12 (83%) patients had a disturbed sleep architecture measured by a PSQI > 5 points. Furthermore, mood disturbances indexed by a BDI score > 12 occurred in 9/12 (75%) patients, with 4/12 (33%) exhibiting at least moderate depressive symptoms (BDI score > 19). All patients presented with BMI values < 30 kg/m^2^ (Table 3).

**Table 3:** Functional state (body weight, sleep, mood, quality of life) and saliva concentrations of oxytocin and IL-1ß at baseline

Patient no	BMI kg/m^2^	Migraine Type	MIDAS score/grade	BDI score	PSQI score	EQ-5D-5L	Oxytocin saliva pg/ml	IL-1ß saliva pg/ml
1	22	CM+	93/IV	25	8	12	61.6	215
2	19	EM+	13/III	2	2	6	27.5	195
3	23	EM−	92/IV	23	10	15	24.1	1000
4	27	EM−	39/IV	20	10	5	29.6	822
5	28	CM+	73/IV	44	13	18	105.3	334
6	20	EM−	40/IV	12	10	8	20.6	194
7	24	EM+	51/IV	7	9	9	16.3	168
8	22	EM−	2/I	9	10	7	41	261
9	28	EM+	47/IV	6	13	7	120.7	1000
10	27	EM+	16/III	12	7	11	16.9	272
11	24	EM+	106/IV	10	19	14	44.2	730
12	19	EM−	17/III	0	4	8	22.7	262

*BDI* Becks depression inventory, *BMI* body mass index, *CM* chronic migraine, *EM* episodic migraine, *EQ-5D-5L* EuroQol five-dimensional five level scale, *f* female, *MIDAS* Migraine Disability Assessment, *PSQI* Pittsburgh Sleep Quality Index

Baseline assessment of the healthy control group (HC) demonstrated similar characteristics compared to the migraine group (14 females; mean age, 46.9 years, ranging from 22 to 59 years, BMI 22.1 ± 1.7).

### Sample collection and laboratory assessment

Saliva samples from patients were collected at a standardized time (8.00–9.00 a.m.) in the morning (at baseline and again after 10 weeks of nVNS) in fasting condition in an inter-ictal interval (defined as 48 h apart from an ictus). Cytokine levels were assessed using high-sensitivity ELISA kits obtained from BD Biosciences Cell Analysis (IL-1β) (Heidelberg, Germany). Saliva samples were collected using pre-chilled Salivettes (Sarstedt, Nuembrecht, Germany). Salivettes were immediately centrifuged at 4180*g* for 2 min and aliquoted samples were stored at − 80 °C until assayed. Salivary OXY concentrations were determined by using a 96 well commercial oxytocin ELISA kit (IBL, Hamburg, Germany). Measurements were performed in duplicate, and samples were treated following kit instructions. According to the manufacturer, the sensitivity limit of the assay is 11.7 pg/ml. The assay’s intra-assay and inter-assay coefficients of variability are 9.1–12.4% and 5.2–14.5%, respectively.

Saliva samples for OXY and IL1ß were obtained from a healthy control group (HC) consisting of 14 females (mean age, 46.9 years; range 22–59 years) matching the demographic characteristics of the treatment group. For reliability reasons two saliva samples per person were measured and the mean value was used for further calculations. Lin’s concordance correlation coefficient for the two saliva samples showed sufficient reliability (r = 0.67, p < 0.01). Healthy controls were recruited from the local population by means of online advertisement, public postings and contacts to assisted living facilities. Subjects were free of any current physical or psychiatric illness as assessed by medical history. After completion of the study, participants received monetary compensation.

### Cervical nVNS stimulation paradigm

Cervical nVNS (gammaCore) received CE-marked approval for the acute and preventive treatment of primary headache disorders (migraine, cluster headache) and medication-overuse headache and was approved by the US Food and Drug Administration for the acute treatment of episodic cluster headache and acute pain associated with migraine.

Patients self-administered bilateral (first right–second left) nVNS therapy twice daily, i.e. each morning and afternoon. Self-stimulation lasted for 120 s. For attack treatment, patients were instructed to administer one additional bilateral application at the onset of each headache attack in conjunction to medication. An appropriate and standardized patient instruction of the nVNS device was supervised from the same instructor at baseline and throughout the entire study period.

The nVNS device was positioned medially to the sternocleidomastoid muscle and laterally to the larynx, with the following stimulation specifications: 1-ms bursts of 5 kHz sine waves, repeated every 40 ms (25 Hz) with an adjustable stimulation intensity (from 0 to 24 V). A conducting gel was applied in order to ensure transdermal signal conductivity.

### Statistical analysis

Normality of the data was assessed by a Shapiro–Wilk test. Normally distributed data are presented as mean ± SEM, while non-normally distributed data are presented as box plots with whiskers showing minimum and maximum values. Pearson’s correlation coefficients were used to assess linear associations between different parameters, while Spearman’s correlation coefficients were determined to assess non-linear associations. A p value < 0.05 was considered significant. The data was analyzed using GraphPad Prism 5.00 (San Diego California, USA).

## Results

### nVNS and migraine-associated head pain severity and frequency

**Fig. 1:**
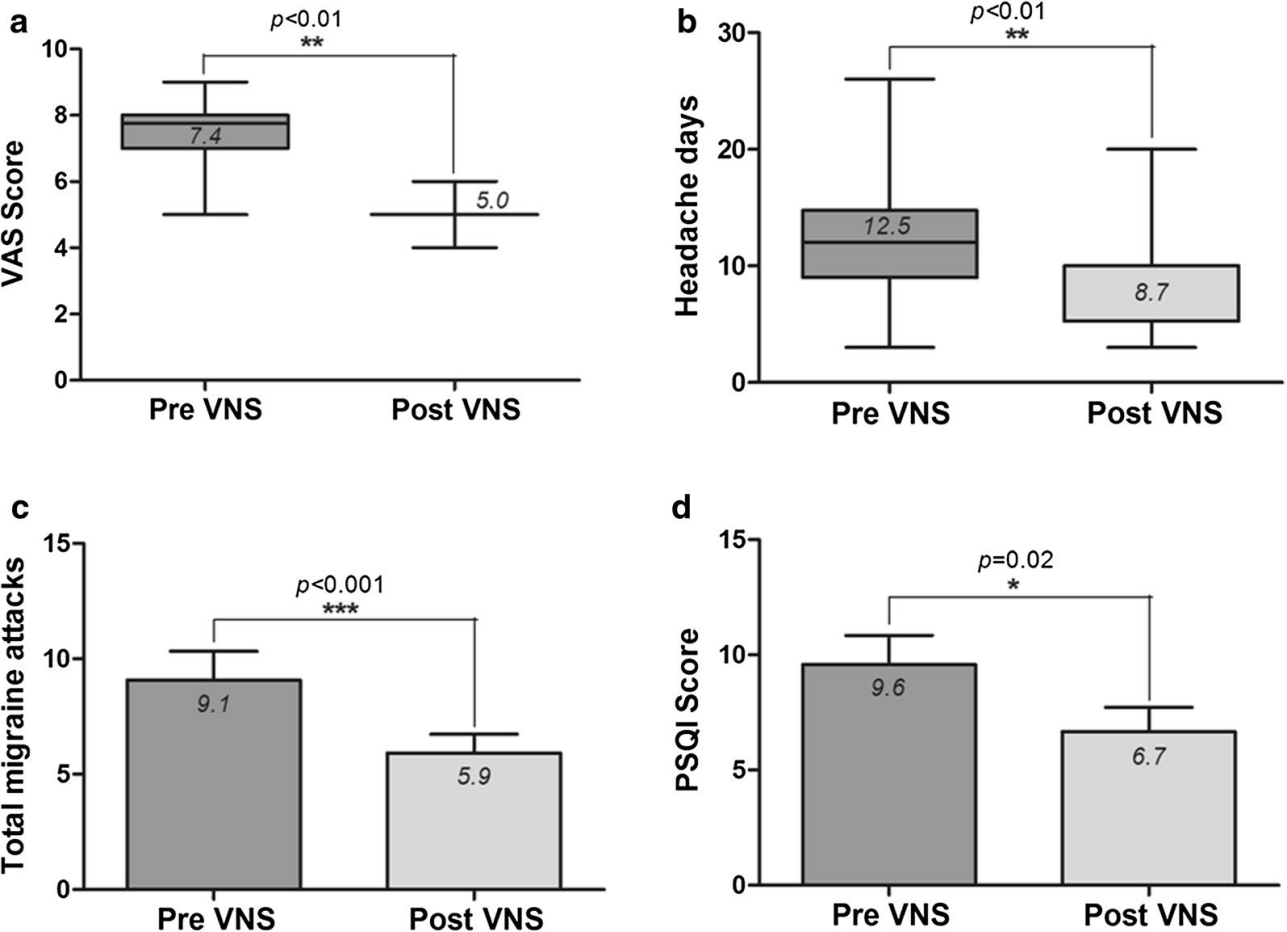
**a**–**d** Pain intensity (VAS) score, migraine frequency (headache days, total number of attacks) and Pittsburgh Sleep Quality Index (PSQI) score at baseline (preVNS) and follow-up (postVNS) in all patients. Mean values with Standard deviations are presented. “*” Indicate the statistical significance

The mean VAS score decreased from 7.4 ± 0.3 at baseline to 5 ± 0.2 (p < 0.01, 95% CI 4.6–5.4) (Fig. 1a). The mean number of headache days in the nVNS group was 12.5 ± 1.7 at baseline and 8.7 ± 1.3 (p < 0.01; 95% CI 5.8–11.5) at the 11th week (end of nVNS treatment), while the number of attacks declined from 9.1 ± 1.5 at baseline to 5.9 ± 0.8 at the end of nVNS therapy (p < 0.01; 95% CI 4.1–7.7) (Fig. 1b, c).

**Fig. 2:**
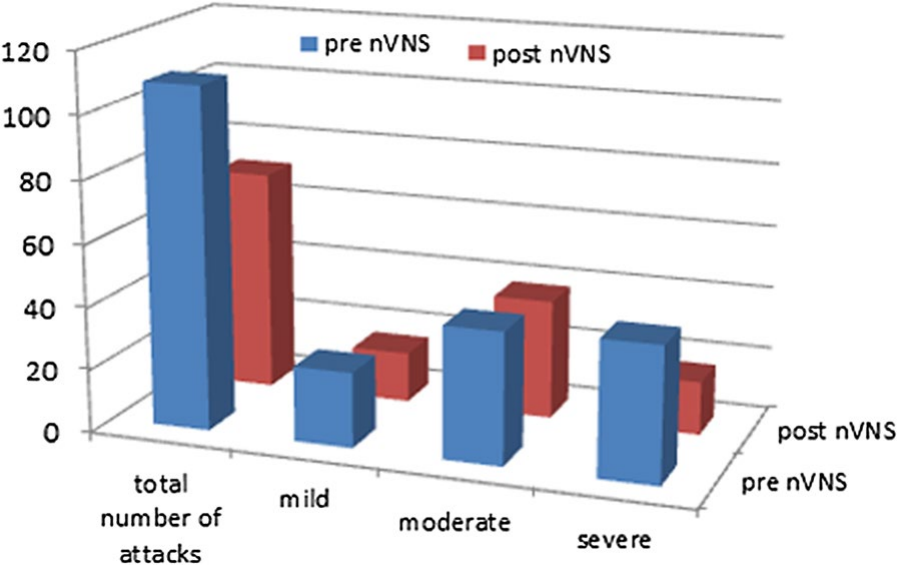
Total numbers of attacks and distribution of mild/moderate/severe rated attack severity at baseline and post nVNS. Mean values with Standard deviations are presented. “*” Indicate the statistical significance

The total number of attacks decreased by 1/3 from 109 (24 mild–42 moderate–43 severe) at baseline to 71 (16 mild–38 moderate–17 severe), with a 60% reduction in the percentage of severe attacks (Fig. 2). At baseline, only one patient achieved pain freedom within 2 h after treatment with acute medication. Following 10 weeks of adjunctive nVNS therapy, the proportion of patients achieving pain freedom climbed to 4.

### nVNS and migraine-associated depressive symptoms, impairment of functional capacity and sleep architecture

Migraine-related abnormalities in sleep architecture as assessed by the PSQI improved after nVNS therapy (9.6 ± 1.3 to 6.7 ± 1.1; p < 0.01; 95% CI 4.3–9.0) (Fig. 1d). Functional capacity measured by the MIDAS score, migraine-associated clinical depressive symptoms quantified by the BDI score and the EQ-5D-5L, however, did not change [BDI (baseline mean 14.2 vs post nVNS 12.6; p = 0.77) and EQ-5D-5L (baseline mean 10.0 vs post nVNS 9.3); p = 0.64] (Table 4).

**Table 4:** Mean values of clinical scores at baseline and post-nVNS treatment given for sleep, mood, migraine-associated disability and quality of life

	Pre nVNS	Post nVNS	p-value
PSQI	9.6	6.7	0.02
BDI	14	12.5	0.77
MIDAS	49	38	0.44
EQ-5D-5L	10	9	0.64

*BDI* Becks depression inventory, *EQ-5D-5L* EuroQol five-dimensional five level scale, *MIDAS* Migraine Disability Assessment, *nVNS* non-invasive vagus nerve stimulation, *PSQI* Pittsburgh Sleep Quality Index

### nVNS effects on saliva concentrations of oxytocin and IL-1ß

**Fig. 3:**
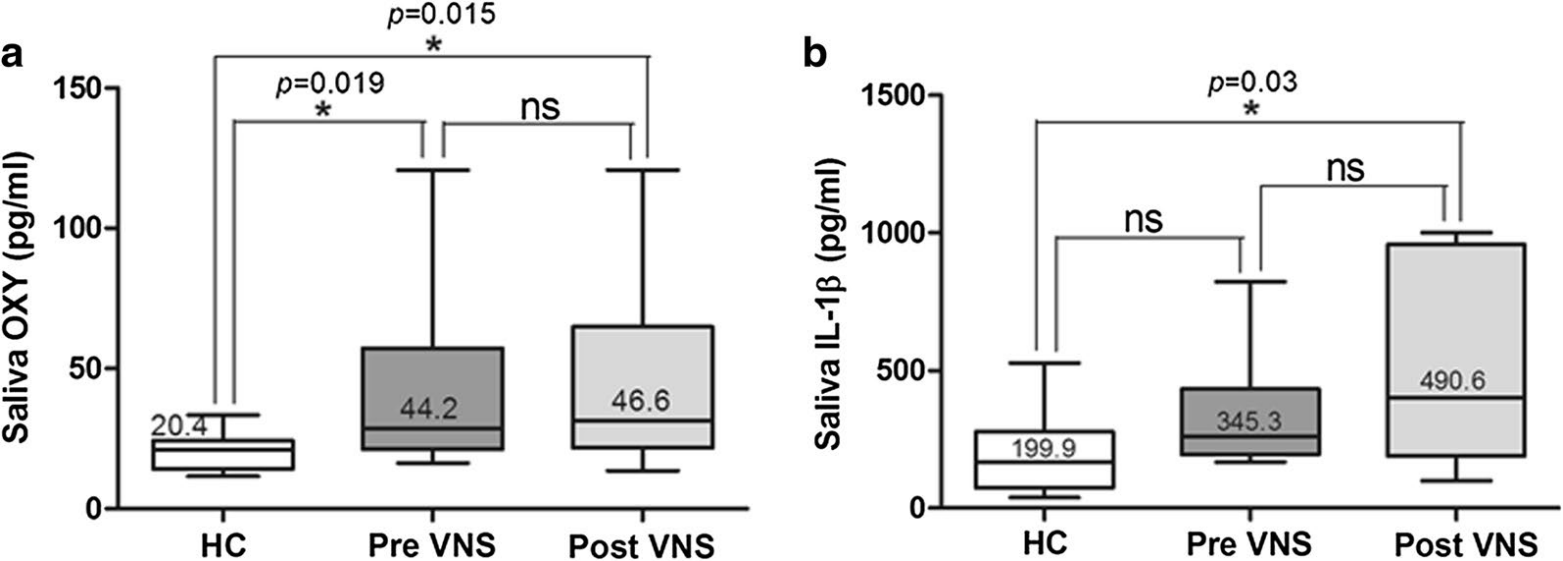
**a**,** b** Oxytocin and Interleukin-1ß analysis (mean values with standard deviation and p-values). Saliva measurements for Oxytocin and IL-1ß at baseline (preVNS) and follow-up (postVNS) are compared to healthy controls. Mean values with Standard deviations are presented. “*” indicate the statistical significance. Abbreviations: *OXY* Oxytocin, *IL*-*1ß* Interleukin-1ß, *HC* healthy controls, *ns* not significant

Pre-nVNS oxytocin levels were more than doubled than those measured in healthy controls (HC: 20.4 ± 1.7 pg/ml vs pre-nVNS patients: 44.2 ± 10.1 pg/ml; p < 0.05; 95% CI 22.1–66.3) and increased without significant difference after nVNS therapy (pre-nVNS patients: 44.2 ± 10.1 pg/ml vs post-nVNS patients: 46.6 ± 12.6 pg/ml) (Fig. 3a). Cytokine saliva levels of IL-1β increased after nVNS therapy, yielding 2.5 times higher values than those measured in healthy controls (p < 0.05) (HC: 199.9 ± 41.4 pg/ml vs pre-VNS patients: 345.3 ± 73.3 pg/ml vs post-nVNS patients: 490.7 ± 113.1 pg/ml; p < 0.05, 95% CI 234.6–746.5) (Fig. 3b).

**Fig. 4:**
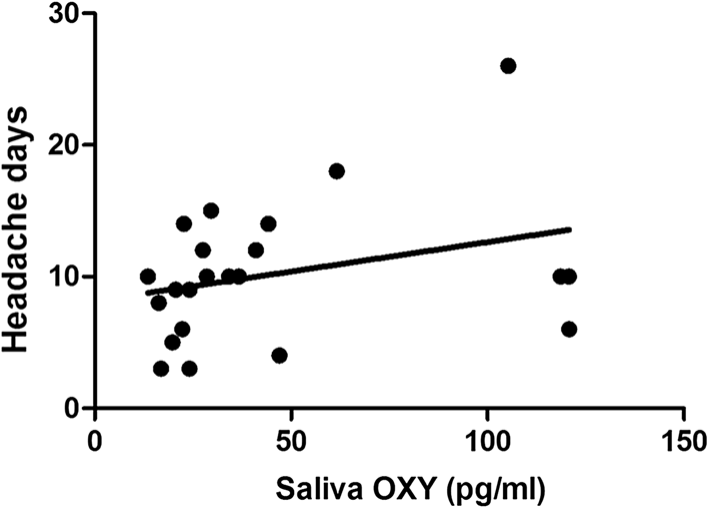
Cumulative correlation analysis between pre-/postnVNS OXY levels and nVNS outcome in headache frequency (headache days/month). Assessment of cumulative pre- and post-nVNS OXY levels showed a trend towards association with the headache days per month (r = 0.379, p = 0.08). *OXY* Oxytocin, *IL*-*1ß* Interleukin-1ß, *HC* healthy controls, *ns* not significant

**Fig. 5:**
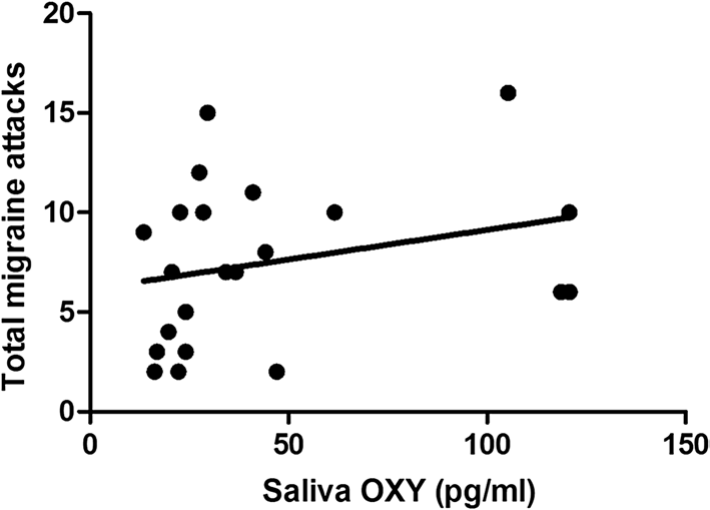
Cumulative correlation analysis between pre-/postnVNS OXY levels and nVNS outcome in headache frequency (attacks/month). Assessment of cumulative pre- and post-nVNS OXY levels showed no association with number of attacks per month (r = 0.343, p = 0.12). *OXY* Oxytocin, *IL*-*1ß* Interleukin-1ß, *HC* healthy controls, *ns* not significant

Assessment of cumulative pre- and post-nVNS oxytocin levels showed a trend towards association with the headache days per month (r = 0.379, p = 0.08) (Fig. 4), but not with number of attacks per month (r = 0.343, p = 0.12) (Fig. 5). By contrast, no significant correlation or trend between global salivary IL-1β and migraine assessment parameters was observed. Prior to nVNS treatment and within the treatment period no clinical systemic disease was observed (CRP below 0.4 mg/dl).

### nVNS associated adverse events

Two patients reported mild treatment-related adverse events (AEs), most commonly skin irritation. No severe or serious AEs occurred.

## Discussion

### Brief summary of study findings and comparison with available literature

Our findings add to pre-existing evidence for the potential therapeutic value and safety of the preventive and acute use of nVNS as an adjunctive to prophylactic and abortive drugs in EM patients. We observed comparable responsiveness for head pain severity and frequency [9–19]. A remarkable reduction in severe attacks was observed in our cohort, similiar responsiveness was observed for pharmacological interventions. Ferrari et al. performed a meta-analysis and reported up to 59% response rates after 2 h (improvement moderate/severe to mild/no pain), 30% pain free state after 2 h and 20% sustained pain free state (no headache recurrence or use of rescue medication 2–24 h after baseline) for acute pharmacological migraine interventions [8]. Most recently, Martelletti et al. assessed additional secondary outcome parameter of the PRESTO study and found a higher rate of pain-freedom and pain-relief attacks as well as a higher rate of severity reduction in migraine patients compared to sham stimulation [17]. This meaningful improvement was accompanied by a considerable rescue medication decrease [18]. However, the antinociceptive head pain potential of the vagus nerve was confirmed in a further t-VNS study targeting the auricular branch at low frequencies (1 Hz) with 30% responder rate (responder defined as ≥ 50% reduction in headache days) [19]. Additionally, our feasibility study demonstrated an improved sleep quality with adjunctive nVNS, which is in line with previously published data [10, 11].

No association was found between those with excellent response compared to less favorable outcome and specific saliva OXY and IL-1β measures. Of note, our study enrolled EM and CM participants, as it has been well documented, that both subtypes encompass different characteristics and beyond doubt have been linked to different pathomechanisms [5–7]. Overweight (BMI 25–30 kg/m^2^) was present in 4 out of 12 patients, thus it cannot be excluded, that this fact may have a considerable impact on the increased inflammatory levels. In contrast to earlier hypothesis restricting the function of white adipose tissue (WAT) as a metabolic storage organ, current revised concepts consider WAT as an inflammatory endocrine active organ with the capability to promote or suppress peripheral and central inflammation via crosstalks between adipocytes (e.g. synthesis of leptin/adipokines) and the innate and adaptive immune system. Obesity as a low-grade chronic inflammation has been associated with tissue hypoxia/necrosis with consecutively upregulation of the pro-inflammatory response via cellular (M1/2 macrophage—Th1/Th2 cells phenotype transformation) and molecular (IL-1β, IL-6, TNF-α) pathways [51]. Hence, future inflammatory migraine research should consider targeting peptides of the adipokine superfamily [51].

Inter-ictal oxytocin and saliva levels were significantly higher in migraine patients compared to healthy controls at baseline and subtly increased after nVNS. So far, most of the reported studies determined cytokine, not oxytocin, in serum and compared inter-ictal versus ictal cytokine signaling. Significantly increased ictal IL-1β, IL-6 and TNF-α serum concentrations were measured in migraine patients with/without aura compared to post-ictal (after 1 week treatment) and healthy subjects not clearly indicative for a predictive value. Similar results were published addressed to migraine and peri-ictal cytokine analysis [38–40]. In our trial, oxytocin and IL-1β screening was performed post-ictally and differed in the choice of the investigated biofluid (saliva). Indeed, it would have been of interest to collect ictal values and additional markers relevant for migraine such as CGRP, but was not performed according to our study protocol. As expected we found higher post-ictal concentrations in migraine patients compared to healthy controls. In line with the findings of Perini et al., it is likely, that ictally assessed saliva concentrations would have displayed higher values compared to our post-ictal results [39]. Nevertheless, elevated saliva levels of inflammatory markers may serve as head pain susceptibility screening tool in migraine patients. After nVNS therapy, both mediators further increased along with an improved head pain state by preventive and abortive means. On the one hand, the increased oxytocin levels may be driven by the observed marked pain relief in our cohort. In several experimental studies, oxytocin has been demonstrated to be released in response to activated sensory neurons of the body (pinch, touch). In particular, electrical stimulation of somatic sensory neurons and afferent fibers of the vagal nerves was shown to effectively increase oxytocin plasma levels immediately after stimulation, which may support the observed increased oxytocin levels in our VNS treated study population [52]. In addition, it is important to note, that 50% of our cohort suffered from migraine-associated aura. Cortical spreading depression as the electrophysiological correlate of aura has been suspected to evoke astrocytes (microglia) induced synthetization and immune response via IL-1β among other cytokines [53]. Other human studies found a correlation between oxytocin concentrations and head pain intensity in migraine, contrary, we observed a trend towards association of migraine frequency (headache days/month) and oxytocin saliva levels [53]. On the other hand and to our surprise, concentrations of pro-inflammatory IL-1β was higher after nVNS compared to baseline. Possible explanations may be the fact that although clinically improved none of the nVNS treated subjects could be classified as head pain free suggesting an ongoing inflammatory process. Importantly, preliminary data indicate that intra-nasally administered oxytocin (32 IU) reduces pain in two patients with chronic migraine headache. This effect was reduced in patients who had taken nonsteroidal anti-inflammatory drugs suggesting that the anti-nociceptive effect of oxytocin is cytokine-dependent [33].

Only one human study assessed possible effects of cervical nVNS on the peripheral components of the neuro-immune reflex in healthy humans. Lerman and colleagues measured healthy individuals randomized to verum and sham cervical nVNS treatment. Chemokine levels assessed at baseline, and at 90 min and at 24 h after treatment showed a decrease in pro-inflammatory IL-1β, IL-8, and TNF-α levels and an increase in anti-inflammatory IL-10 levels, indicating that nVNS may inhibit pro-inflammatory cytokine release [41]. Other human studies conceptualized to determine peripheral inflammatory profiles of subjects treated with surgically implanted invasive VNS (iVNS) were limited to depression and focal seizure with limited interpretations due to the uncontrolled study design [36, 37]. However, comparable cytokine/chemokine data under VNS “off”-stimulation remain an open question.

### The impact of oxytocin on trigemino-nociceptive signaling

Recently, a preclinical study determined oxytocin receptor expression and co-localization with calcitonin gene-related peptide (CGRP) in the trigeminal ganglion. Application of painful, facial electrocutaneous stimulation and adjunctive capsaicin-driven inflammation increased oxytocin expression in CGRP-containing trigeminal ganglion neurons, indicating the important role of oxytocin in migraine pathophysiology [34].

The relationship between migraine and oxytocin was under investigation in a sophisticated experimental setting including measurement of electrophysiological (TNC firing response) and gene expression (C-fos) parameters after intranasal oxytocin administration compared to placebo [35]. In the first, increased TNC firing rates after electro-cutaneous stimulation of the face were recorded and of note, attenuated by oxytocin, while in the second an increased C-fos expression in TNC neurons was observed after intra-peritoneal injection of nitroglycerin, which was revised in the oxytocin pre-treated group. In a next translational step, intranasal oxytocin was assessed in sham-controlled trials in EM and CM patients [35]. Although no significant differences were observed after 2 h treatment for both migraine subtypes, the CM subgroup demonstrated a trend in favor of the verum treatment. Additional human trials indicate a stronger effect of oxytocin on frequency, rather than on severity. Interestingly, NSAID use was suspected to interfere with oxytocin effects by inhibiting cytokine synthesis [35]. NSAID as an adjunctive rescue medication was present in 5 out of 12 patients in our study.

Migraine as a multi-network brain disorder displays altered sensory (nociception), cognitive, affective and circadian-dependent autonomic features (sleep, metabolism, thermoregulation), of which each component is able to drive head pain onset/attacks [54–56]. Apart from its ictal functions, inter-ictal endogenous oxytocin has been linked to central sensitization (hyperalgesia, allodynia) and neurogenic inflammation in migraine pathophysiology. Such elevated inter-ictal oxytocin concentrations may reflect modulation of extracephalic pain perception and affective distress symptoms. Indeed, it would have been of great interest to quantify these inter-ical appearing clinical features relevant for migraine [56]. Taken the complex and dynamic nature of migraine into account, the authors speculate, that molecular profiling of oxytocin may have a diagnostic and therapeutic potential for migraine-associated symptoms outside the ictal phase [54, 55].

### Pro-inflammatory IL-1 β associated effects in trigemino-nociceptive traffic

Zhang et al. recorded TG neuron response of meningeal nociceptors after local application of IL-1β and IL-6 on dural nociceptors and found that IL-1β, but not IL-6 was able to promote increased activity of TG neurons accompanied with increased mechano-sensitivity of intracranial nociceptors (meningeal afferent signaling) measured von Frey filaments [42].

IL-1β has been suspected to induce upregulation of cyclo-oxygenase 2 mRNA (COX-2) expression in glial cells and neurons of the trigeminal ganglion (TG). These COX-2 dependent pathways lead to prostaglandine release from glial and neuronal TG cells, which in turn stimulates solely neurons of the TG to immediately (1 h after stimulation) produce CGRP, contrary to IL-1β, which demonstrated a delayed CGRP release pattern (24 h after stimulation) suggesting a glia-neuron interaction in the TG [57]. Methylprednisolone reversed the IL-1β effects, but demonstrated no impact on prostaglandine induced CGRP release [58]. Leptin, a metabolic marker produced by WAT cells, has been shown to interact with the COX-2 dependent pathways via crosstalks with IL-1β in glial cells and neurons of the hypothalamic-pituitary axis [59].

The development of acute head pain has been associated with primary afferents activation of the TG driven by dural nociceptors, which are connected with the trigeminal nucleus caudalis (TNC). The TNC itself projects to the trigemino-cervical complex (TCC), and receives reciprocal input from the brainstem, the medulla oblongata, the hypothalamus-pituitary-axis, the thalamic nuclei (intralaminar nucleus of the thalamus) and cortical associated networks.

On the other hand, it has been well described, that the afferent properties of the vagus nerve project via the ncl. tractus solitarii to the locus coeruleus (LC), the dorsal raphe nucleus, the parabrachial plexus, the paraventricular nucleus of the hypothalamus and maybe directly to the TNC and the cervical spinal cord (trigemino-cervical complex, TCC). In view of the anatomic reciprocal connectivity of the vagus nerve, it may be reasonable, that cervical nVNS may impact trigeminovascular nociceptive signaling of pro- and anti-inflammatory markers in different biofluids (plasma, saliva, cerebrospinal fluid) [20, 21, 26–31, 34, 35, 52, 57–59].

Inhaled (olfactory nerve) and/or ingestive (trigeminal nerve) chemical irritants have been suspected to promote pro-inflammatory mediators and to trigger neurogenic inflammation in a broad range of respiratory (asthma) and neurological disorders such as migraine. A dynamic and complex interplay between local (e.g. substance P, bradykinin) and distant efferent effects (adrenergic, cholinergic) characterizes in part the host response, in which mast cell derived immunomodulation plays a pivotal role. Hence, it cannot be excluded, that environmental factors may have an impact on inflammatory phenotyping and quantification of peripheral markers of the neuro-immune axis [60, 61].

In particular, immunomodulatory mast cells (high affinity receptor FceR1) are capable to interact with the innate and adaptive immune response and poses an important role in the genesis of acute/chronic inflammation associated disorders (e.g. migraine). Immunglobuline E (Ig E), Toll-like receptors, IL-1β and IL-36 are known to activate mast cells and to drive a pro-inflammatory state, while IL-37 and IL-38 (member of the IL-1 cytokine family) act as an inhibitor of inflammation. Gallenga and colleagues reported that IL-38 is able to bind on the IL-36 receptor, which in turn blocks mast cell activation [62]. It is important to note, that mast cell response occur in a time dependent manner with an immediate secretion and a delayed synthesis/release of inflammatory active peptides in order to establish a physiological host response. Among the mentioned IL-1 cytokine family, IL-1β increases IL 33 and TNF-α synthesis derived from mast cells. Furthermore, IL-33 interacts with monocytes and promotes mast cell differentiation, maturation and degranulation with subsequent secretion of pro-inflammatory cytokines/chemokines. Contrary, IL-37, an anti-inflammatory member of the IL-1 cytokine family, has the potential to counterbalance the IL-1β evoked innate and adaptive immune response. Therefore, the therapeutic anti-inflammatory value of IL-37 induced blocking of mast cells deserves further clarification in migraine research [63, 64].

### Limitations and future prospects for molecular inflammatory profiling in migraine

This study has several limitations including the uncontrolled design, the small-scale study cohort and a relatively short-term observation period. The main issue is related to the lack of a sham stimulation group (stimulation off) in order to discern what was due to patient’s expectation (treatment self-responsibility) in contrast to the real effects of nVNS. With respect to migraine as a complex brain disorder, such expectation associated with a novel device (like nVNS in our study) may represent a confounder. Different dosages of migraine drugs (preventive/abortive) administered in each patient may represent a confounder. Furthermore, cytokine levels may vary and depend on pre-analytic variables including sample processing, environmental factors, intra- and interindividual variability. The cytokine analysis performed in our study did not consider the dynamic nature of neuro-inflammation nor the circadian neurobiology, thus repetitive measurements are recommended in future trials. The role of the brain-immune-communication in primary headache disorders is of major interests. To this end, immune-phenotyping of migraine is in the beginning. Future research in this field should seek to investigate which immune pathways are overactivated in migraine, how immune cell hyperactivity is linked to disease susceptibility, and how environmental and genetic factors influence immune activation and disease manifestation. Immune-phenotyping should consider cell profiling in several flow cytometry panels and whole blood stimulation assays with a range of innate immune stimuli. Can we use molecular profiling to predict and individualize neurostimulation (nVNS) therapy? Can we target cytokines as diagnostic tools? Currently the answer is no as the precise mechanisms of the neuro-immune communication in migraine pathophysiology remains unclarified. Alternatively, advanced statistical methods capable to establish categorical-based dimensions may support the potential and the integration of biobank-based immune-phenotyping, thus help to define migraine specific characteristics and subsets (biotypes) of patients more or less likely to respond to neurostimulation therapy [65, 66]. However, this study firstly approached to screen oxytocin and IL-1β in saliva and attempted to proof the feasibility of saliva analysis and undoubtedly, but was biased by the uncontrolled study design.

## Conclusion

Ten weeks of adjunct nVNS therapy significantly decreased migraine severity and frequency in patients with EM (with/without aura). In addition, migraine-associated sleep impairment was improved. Inter-ictal saliva concentrations of oxytocin and IL-1β were significantly higher in migraine patients compared to healthy controls. An evidence-derived conclusion about the predictive value and usefulness of saliva assays is clearly limited by the provided uncontrolled study design. Clearly, our findings along with preclinical and human available literature data point to the necessity to monitor changes in a broad array of anti- and pro-inflammatory markers during nVNS since treatment effects may be reflected in altered ratios of different markers. Thus, the observed salivary levels (and nVNS-induced changes) must be interpreted with caution and currently remain at an experimental stage (feasibility). However, the assessment of cytokine/chemokine plasma and/or saliva levels addressing the pathogenesis and treatment of migraine may represent a novel approach and may be worthy for being re-visited under controlled study condition in order to evaluate its usefulness beyond subjective patient´s self-report.

## Abbreviations

EVENT: chronic migraine prevention with non-invasive vagus nerve stimulation
PRESTO: prospective study of nVNS for the acute treatment of migraine
PVN: paraventricular nucleus
SOP: supraoptic nucleus
TNC: trigeminal nucleus caudalis
TCC: trigemino-cervical complex
TG: trigeminal ganglion
LC: locus coeruleus
VNS: vagus nerve stimulation
IL: interleukin
TNF: tumor necrosis factor
CGRP: calcitonin gene-related peptide
COX: cyclooxygenase
WAT: white adipose tissue
EM: episodic migraine
CM: chronic migraine
ICHD: International Classification of Headache Diagnosis
PSQI: Pittsburgh Sleep Quality Index
EQ-5D-5L: Euro quality of life scale
MIDAS: migraine disability score
BMI: body mass index
